# Growth of U-Shaped Graphene Domains on Copper Foil by Chemical Vapor Deposition

**DOI:** 10.3390/ma12121887

**Published:** 2019-06-12

**Authors:** Ming Pan, Chen Wang, Hua-Fei Li, Ning Xie, Ping Wu, Xiao-Di Wang, Zheling Zeng, Shuguang Deng, Gui-Ping Dai

**Affiliations:** 1 Key Laboratory of Poyang Lake Environment and Resource Utilization, Nanchang University, Ministry of Education, School of Resources Environmental & Chemical Engineering, Nanchang University, Nanchang 330031, China; mpan@email.ncu.edu.cn (M.P.); c.wang@email.ncu.edu.cn (C.W.); pwu@email.ncu.edu.cn (P.W.); 402531318006@email.ncu.edu.cn (X.-D.W.); zlzengjx@ncu.edu.cn (Z.Z.); 2 Institute for Advanced Study, Nanchang University, Nanchang 330031, China; hfli@email.ncu.edu.cn (H.-F.L.); nxie@email.ncu.edu.cn (N.X.); 3 School for Engineering of Matter, Transport and Energy, Arizona State University, Tempe, AZ 85287, USA

**Keywords:** graphene, U-shaped, CVD

## Abstract

U-shaped graphene domains have been prepared on a copper substrate by chemical vapor deposition (CVD), which can be precisely tuned for the shape of graphene domains by optimizing the growth parameters. The U-shaped graphene is characterized by using scanning electron microscopy (SEM), atomic force microscopy (AFM), transmission electron microscopy (TEM), and Raman. These show that the U-shaped graphene has a smooth edge, which is beneficial to the seamless stitching of adjacent graphene domains. We also studied the morphology evolution of graphene by varying the flow rate of hydrogen. These findings are more conducive to the study of morphology evolution, nucleation, and growth of graphene domains on the copper substrate.

## 1. Introduction

Graphene, an ideal 2D versatile carbon material with a combination of the honeycomb-like arrangement, has drawn extensive attention in the research field, due to its good properties, such as high carrier mobility [1], mechanical tensile strength [2], and thermal conductivity [3]. Graphene has been prepared in several different ways since Geim et al. first obtained a single-layer polycrystalline graphene by the mechanical stripping of highly oriented pyrolytic graphite [4], such as the exfoliation and chemical reduction of graphene oxide [5,6,7], epitaxial growth of SiC [8], and by depositing on metal using chemical vapor deposition (CVD) [9,10]. In the method above, graphene growth on metal by CVD is regarded as the most promising and cost-effective way to synthesize graphene for experimental research and industrial development, owing to its convenience, reproducibility, and high controllability. Among the metal substrate used for the synthesis of graphene by CVD, nickel (Ni) [11] and copper (Cu) [9] are the most used. In the meantime, the fabrication of uniform and large-area graphene on copper foil is the most promising synthesis method because of the solubility of the carbon atom in a Cu substrate when compared to a Ni substrate [9,12]. 

Nowadays, on the one hand, using the CVD method for preparing large-area graphene on a copper foil surface is a synthetic way with great potential for industrial development, but the growth of graphene is accompanied by crystal imperfections and boundaries [13,14]. This has a great influence on its properties and provides an obstacle to the formation of a high-quality single-layer graphene, and this has brought great difficulties to its research and application. Therefore, how to expand and seamlessly merge graphene domains into a large-sized defect-free graphene sheet has become an important research topic. On the other hand, several odd shapes of graphene can be prepared on a copper foil by CVD and by regulating growth parameters, including hexagons [15,16,17,18,19,20], four-lobed [21,22], triangular [23,24], rectangular [25,26,27,28], pentagonal [29], and 12-pointed [30], which contributes to an understanding of the nucleation and expansion mechanisms of graphene grains to some extent. In addition, how to grow high-quality single-crystal graphene by optimizing experiment conditions is still the focus of both the academic field and industrial application. Thus, it can reduce surface defects by either chemical or physical processes, or by prolonging the annealing time to pretreat the metal surface, which is beneficial to the growth of the graphene on the metal surface. Van et al. [31] reported that the surface of a copper foil was pretreated by chemical-mechanical polishing in order to reduce the roughness and defects of the Cu foil, which was later used as a substrate for the growth of a single-crystal graphene by CVD. During that process, they obtained a large-area graphene film by the seamless stitching of the graphene grains to the regular hexagonal graphene domains. Wang et al. [27] found that the decrease of the nucleation density, the reduction of defects, and the contamination on the Cu surface after a prolonged annealing time in a hydrogen environment, obtained a high-quality single-layer rectangular graphene and continuous graphene films on the substrate. Geng et al. [29] investigated whether liquid Cu could eliminate the effects of solid copper grain boundaries, and obtained uniform, single-domain, high-quality hexagonal graphene flakes by CVD. Their works are advantageous for studying the nucleation growth of graphene and forming a continuous graphene film. These studies have also demonstrated the great potential in graphene morphology evolution and the formation of a high-quality large-sized graphene film. Therefore, studying the morphology evolution of graphene is still very significant in order to understand the nucleation growth and expansion mechanism of graphene.

In this work, we report for the first time the growth of large-sized “U” shaped graphene domains on a solid-Cu substrate by using ambient pressure CVD. These U-shaped graphene domains also run parallel to each other as well as along the gas flow direction. Evidently, the U-shaped graphene has a smooth edge, which is of importance to seamlessly stitching of the adjacent graphene domains. Moreover, the U-shaped graphene size could change when the methane flow rate was adjusted, and the morphology of the graphene domain could be different when the hydrogen flow rate was adjusted.

## 2. Experimental Section

### 2.1. Synthesis of Graphene Domains by CVD

**Figure 1:**
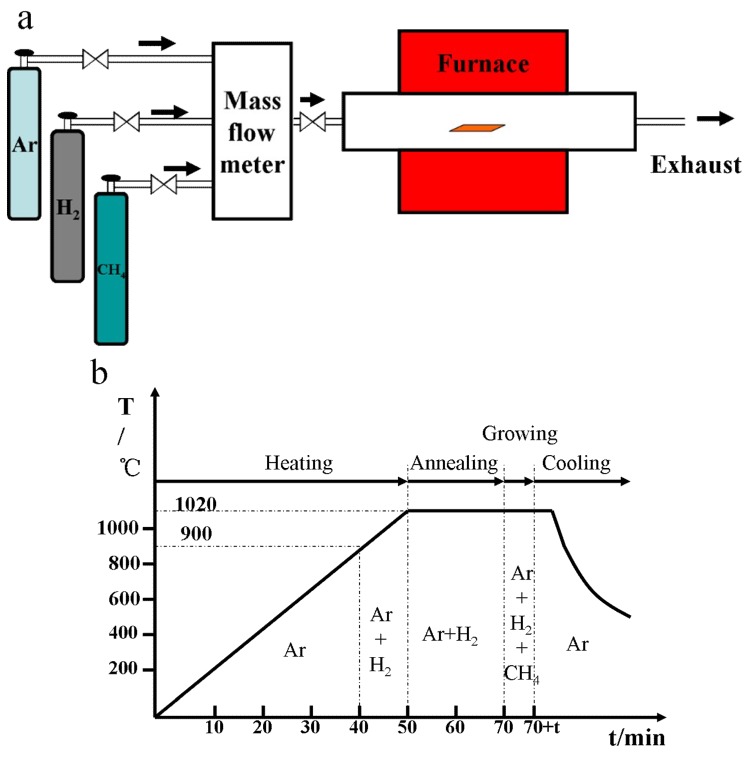
(**a**) Schematic illustration of the chemical vapor deposition (CVD) synthesis of graphene. (**b**) The process of graphene growth by CVD (t = 90 s).

An atmospheric pressure CVD system was used with a quartz tube which was 6.5 feet in length and has an inner diameter of 2 in. as illustrated in Figure 1a. Cu foil with a thickness of 25 μm (Alfa Aesar 13382, 99.8% pure, Alfa Aesar, Haverhill, MA, USA) was used as a growth substrate, which was loaded into a chamber after its surface was pretreated. First, Cu foil was soaked in dilute nitric acid for several seconds after it was immersed in acetic acid and deionized water for 3 min, respectively. The Cu foil was then dried with N_2_ and the clean Cu foil was placed into the quartz tube. The Cu foil was heated to 900 °C under a flow rate of 100 standard cubic centimeters per minute (sccm) for Ar, and 75 sccm for H_2_. After being annealed for 20 min at 1020 °C, CH_4_ with a flow rate of 20 sccm was introduced to the chamber for 90 s, then the flow of CH_4_ and H_2_ are turned off while maintaining a flow rate of 100 sccm for Ar, and the reactor is quickly moved to the other side to bring the temperature of the reaction zone down to room temperature. The whole process is illustrated in Figure 1b.

### 2.2. Transfer of Graphene

The graphene-covered Cu substrate surface was spin-coated with a polymethylmethacrylate (PMMA). After 2 h, the Cu foil was etched by using a 0.5 M ammonium persulfate solution. In the meantime. The PMMA/graphene was transferred to deionized water for the removal of chemical residues. Then, PMMA/graphene was pasted on to a target substrate and then immersed into acetone to remove the PMMA layer. Finally, the substrate was placed in a vacuum drying oven and then dried by heating.

### 2.3. Graphene Characterization

Scanning electron microscopy (SEM) (FEI Quanta200F, Waltham, MA, USA), atomic force microscopy (AFM) (Agilent 5500, Santa Clara, CA, USA), Raman spectroscopy (Horiba HR Evolution, Irvine, CA, USA) with a 100× lens, and transmission electron microscopy (TEM, JEOL 2010F, Peabody, MA, USA) were used to characterize the graphene film.

## 3. Results and Discussion

**Figure 2:**
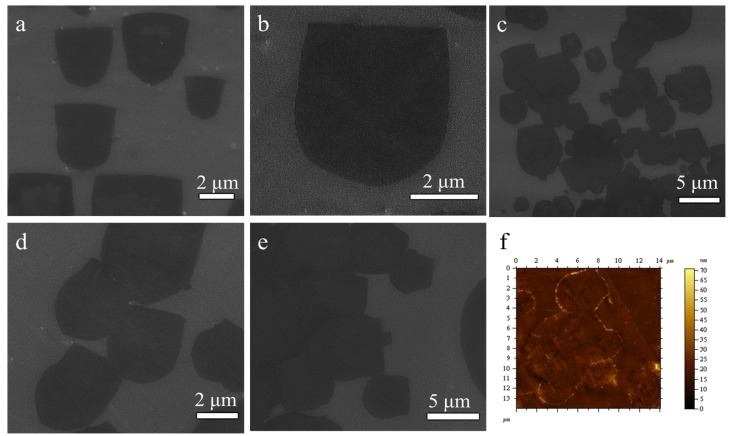
(**a**) SEM image of the U-shaped graphene domains on the copper foil. (**b**) The magnified SEM image of an individual U-shape graphene. (**c**,**d**) SEM images of the coalescence of the different graphene domains. (**e**) A SEM image of the continuous graphene film. (**f**) The atomic force microscopy (AFM) image of the merging graphene domains.

As illustrated in Figure 2a, we successfully synthesized large-sized U-shaped graphene domains at an appropriate growth time by introducing a higher CH_4_ flow rate in an ambient pressure CVD on Cu foil, which was never reported before. Each U-shaped graphene domain has the same growth direction, the domains are parallel to each other, and the domain direction is consistent with the reaction’s gas-flow direction. The graphene domains display differences in size, indicating the nucleation and growth of graphene domains are different. Considering the total gas flow rate was relatively high in the reactor, it is possible that the reaction gases were in an unstable condition, which is difficult to achieve an even distribution of carbon species in such a short growth time [26]. Therefore, while the simultaneous nucleation of graphene domains, the partial graphene domains growth was limited due to the insufficiency of local carbon species. The magnified SEM image of an individual U-shaped graphene is further shown in Figure 2b, and it shows a perfect U-shaped graphene with a smooth edge, which facilitates seamless stitching between graphene domains.

As shown in Figure 2c, it can be seen that many U-shaped graphene domains merge with each other to form a large domain, which shows the different stages of the merging process of the graphene domains with various sizes, and the nature of the U-shaped graphene domain is still obvious. The SEM image shows the U-shaped graphene domains were merged together in two different ways, namely straight-edge merging with the straight one, and straight-edge merging with the curved one. Additionally, it shows that the two ways have no effects for the formation of a larger-sized graphene domain during the merging process. Additionally, the adjacent U-shaped graphene domains were merged to each other if they were close enough. As shown in Figure 2d, the SEM image shows the merging process in the early stages for the U-shaped graphene domain. The adjacent U-shape graphene domains were coalesced into a large-area graphene domain as shown in Figure 2e when the growth time was increased. In general, the nucleation and growth of the graphene domain depended on the saturation concentration of the activated carbon species on the copper surface [22]. When the growth time was increased, the methane continued to decompose, resulting in the oversaturation of the carbon species on the copper surface. Moreover, the carbon atoms can be continuously driven to the edge of the domain, which then caused the graphene to continue to grow. As illustrated in Figure 2f, the AFM image shows the merging process of several adjacent graphene domains in the early stage, which demonstrates the seamless coalescence for the adjacent graphene domains. 

**Figure 3:**
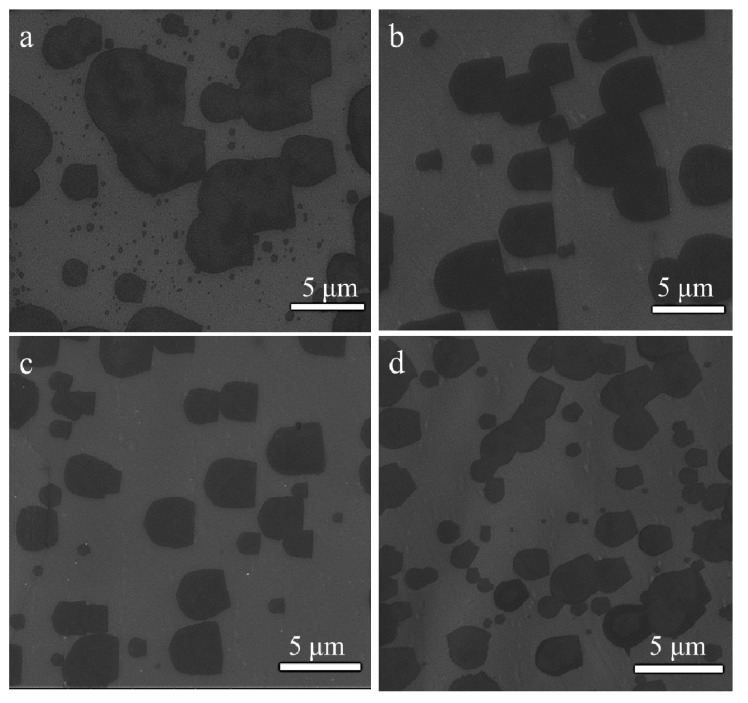
(**a**–**d**) SEM images of the growth of U-shaped graphene under different methane flow rates (20, 25, 30 and 35 sccm, respectively).

The formation of the graphene domain is closely related to the experiment parameters [17], such as the methane flow rate. As shown by the SEM images in Figure 3, the U-shaped graphene domains grew under different flow rates of methane, while other parameters were kept constant. In addition, the domain size decreased and domain density increased when the methane flow rate increased (Figure 3a–d corresponding to 20, 25, 30 and 35 sccm, respectively). In general, when the CH_4_ flow rate increases, more methane molecules can be absorbed on the copper surface and are decomposed to create more free carbon atoms, and therefore the number of possible nucleation sites increases, resulting in the shortage of carbon species around the edges of the graphene domains, which limited the growth in size for an individual graphene domain in our case. 

**Figure 4:**
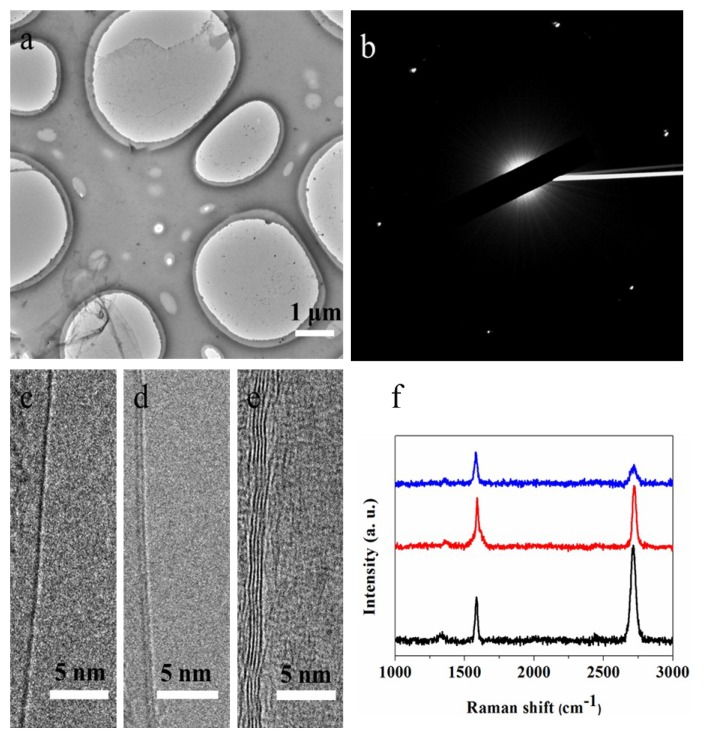
(**a**) TEM image of the graphene domain on the TEM grid. (**b**) Selected-area electron diffraction (SAED) pattern showing a single-crystalline nature of the U-shaped graphene domain. (**c**–**e**) TEM images of the layer number of graphene. (**f**) Ramen spectra of graphene domain on copper foil, showing monolayer and few-layer graphene on copper foil.

In order to further study the layer number and the defects of graphene, the graphene was transferred to a copper grid for TEM observation. As shown in Figure 4a, the graphene domain was successfully transferred to a TEM grid from the copper foil. The structural damage and wrinkles of graphene were observed in TEM images because it was difficult to maintain the original morphology of the film during the transfer process. Besides, the dots on graphene film may be PMMA residues that were not completely dissolved [26]. As shown in Figure 4b, the selected-area electron diffraction (SAED) pattern shows a hexagon diffraction-pattern characteristic, indicating the single-crystalline nature of graphene domain. Meanwhile, the layer number of graphene exhibited in Figure 4c–e has been observed along the edge regions of the film under a high-resolution magnification, showing that the synthesized graphene domains have a single layer and a few layers. In addition, we also characterized the graphene domains by Raman spectra. As shown in Figure 4f, we learned that the Raman spectra show the two prominent Raman peaks of graphene, which locate at ~1591 cm^−1^ and ~2691 cm^−1^, corresponding to the G-peak and the 2D peak, respectively. Moreover, the weak D-peak (~1360 cm^−1^) in the Raman spectra indicate the high quality of graphene film. The I_2D_/I_G_ ratio is about 2.2 for the black curve, which indicates a monolayer graphene region. For the red curve the I_2D_/I_G_ ratio is about one, and for the blue curve the I_2D_/I_G_ intensity ratio is below one, indicating the few layer region of graphene. 

**Figure 5:**
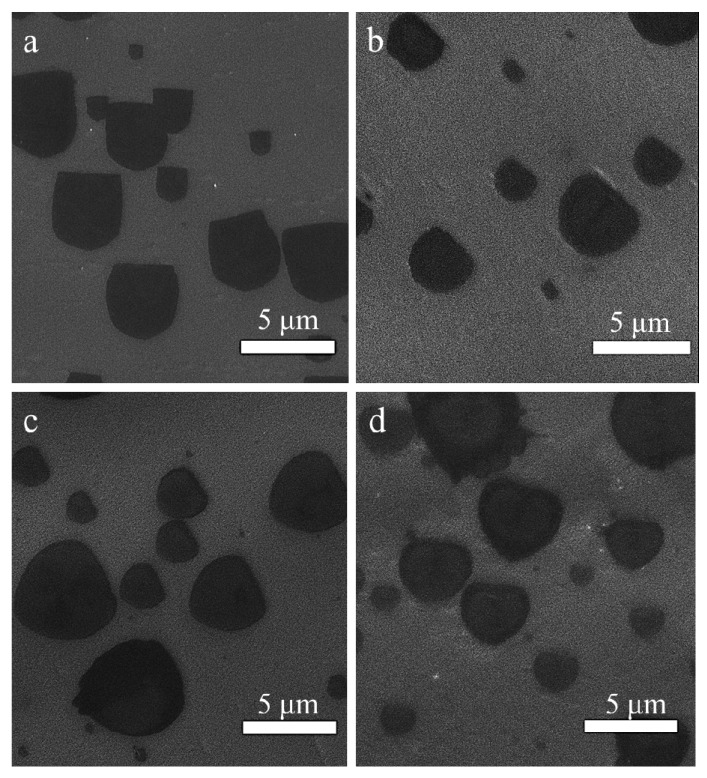
(**a**–**d**) SEM images depicting the evolution of the graphene domain growth on the copper foil at different hydrogen flow rates by atmospheric pressure CVD (75, 65, 60, and 55 sccm, respectively).

In CVD, the evolution of the graphene domain shape is related to many growth conditions, including the surface treatment of substrate, the flow rate of gas, the annealing time and so on. In this work, we found that the hydrogen flow rate can significantly influence the final shape of graphene domains. A series of graphene domains with different shapes are formed on a copper foil at a high flow rate of methane (20 sccm) and a short growth time (90 s) by varying the hydrogen flow rate as shown by the typical SEM images in Figure 5a–d. The U-shaped graphene domains with smooth edges can be successfully synthesized on the copper surface at a hydrogen flow rate of 75 sccm as shown in Figure 5a, and the U-shaped graphene domains are highly reproducible. When we decreased the hydrogen flow rate from 75 sccm to 65 sccm, the morphology of the graphene domain changed. The semi-circle shaped graphene domains (Figure 5b) formed on the copper surface with a smooth semi-circle edge. However, when the hydrogen flow rate was further adjusted to 55 or 60 sccm, the heart-shaped graphene domains (Figure 5c,d) were synthesized. Remarkably, we could clearly observe that, when the hydrogen flow rate varied, the previous relatively stable structure changed, which resulted in the formation of the different graphene shapes as shown in Figure 5a–d, confirming that the morphology of graphene domains can be tuned in a controllable way. Besides, the graphene domains have the same growth direction and are paralleled to each other, which may be beneficial for the growth of a high-quality graphene sheet. In general, the morphology of graphene domains could be predicted from thermodynamics by Wulff construction or kinetic analysis [32]. The morphology and structure of graphene domains have a great influence on graphene properties [17]. The morphology evolution in our case may provide a new way to get different graphene domain shapes by CVD.

**Figure 6:**
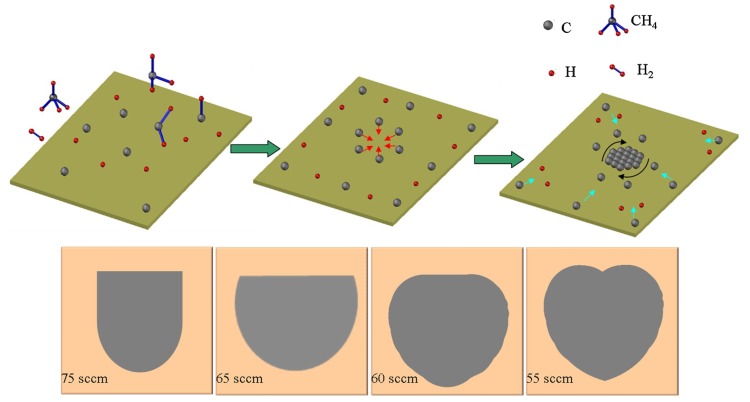
Schematic illustration shows the morphology of the graphene domains on copper foil in different hydrogen flow rates during CVD.

It is possible that the morphology evolution of the graphene domain is caused by the destruction of the dynamic equilibrium mechanism [17]. Therefore, understanding the various graphene domain shapes in the initial stages is very important for understanding the growth mechanism of graphene. Generally, methane molecules are dissociated into carbon radicals and free carbon atoms on the copper surface, and the free carbon atoms aggregate into a nucleus (the red arrow) to form graphene as shown in Figure 6. The diffusion of free carbon atoms on the copper surface has two directions: free carbon atoms diffusion along the nucleation center (the black arrow) and free carbon atoms surface diffusion (the indigo arrow). The competition between two diffusions determines the final morphology of the graphene domain [17]. A compact structure is formed when the free carbon atom finds a favorable site along the nucleation center before other free carbon atoms migrate by the surface diffusion to join it. That is to say, the free carbon atoms near the nucleation center can adhere to the island edge when the surface diffusion rate of free carbon atoms is not large enough, thus forming a compact graphene domain. In addition, hydrogen is one of the key factors in the growth of graphene [26]. It not only acts as an etchant, but it also promotes the dissociation of methane to form free carbon atoms and activate the copper surface to promote the bonding of carbon atoms. However, the high flow rate of hydrogen gas is unfavorable for the dissociation of methane molecules, leaving more undissociated methane molecules on the copper surface, resulting in an energy barrier, which reduces the surface diffusion rate of the free carbon atoms [33], thereby the growth of the graphene domain is suppressed. Simultaneously, during the graphene growth, the high flow rate of hydrogen promotes the etching of the graphene domain. Thus, the U-shaped graphene domain is formed at a high hydrogen flow rate (75 sccm) in our case. With the hydrogen flow rate decreasing, the energy barrier also reduces, resulting in the increasing of the surface diffusion rate of free carbon atoms, and thus making it unstable from its original equilibrium state, so some unique graphene morphologies [18,26] are formed, for example Heart-shaped characterization in our case. It should be mentioned that the process of synthesizing graphene domains by CVD involves complex physical and chemical reactions. Thus, despite this investigation is conducted on the morphology control and evolution of the graphene domain, the detailed accurate mechanism of nucleation and growth of graphene remains unclear, which requires to be further studied.

## 4. Conclusions

In summary, we have controlled the growth of U-shape graphene domains on a copper foil by using CVD at atmospheric pressure. The change in methane flow rate has a great influence on the nucleation density and the size of the U-shaped graphene domain. Besides, different domain morphologies of graphene have been formed, such as semi-circle-shaped graphene and heart-shaped graphene, through varying the flow rate of hydrogen, which indicates the important role of hydrogen in the morphology evolution of graphene domain. This study may provide a new way to fabricate the novel morphology of graphene domains and may contribute to an understanding the growth mechanism of graphene.
